# Cases Illustrative of the Morbid Appearances Which Occasionally Take Place in the Intestines during the Progress of Fever

**Published:** 1829-04-01

**Authors:** 


### CASES ILLUSTRATIVE OF THE MORBID APPEARANCES WHICH OCCASIONALLY TAKE PLACE IN THE INTESTINES DURING THE PROGRESS OF FEVER. By *Richard Bright,* M.D. Physician to Guy's Hospital.

OUR readers are aware that, in examining foreign works and journals, especially the writings of Broussais, Andral, Bretonneau, &c. we frequently drew their attention to the highly important subject of inflammation and ulceration of the mucous membrane of the intestines, as the cause or the consequence of fever. Dr. Hewett, in this country, and several of the hospital physicians in Dublin, have zealously prosecuted this interesting inquiry—and, last of all, we have to introduce to the notice of our brethren, the investigations of Dr. Bright, as set forth in his splendid work—too dear, alas! for the great majority of the profession.

Dr. B. informs us that, during the months of October, November, and December, 1826, there was a considerable influx of fever cases into Guy's Hospital. The disease was not severe, and only required a mild and uniform treatment, with several marked exceptions. The symptoms of abdominal derangement prevailed in the majority of cases, and the stomach was often so irritable as to preclude food and physic. The matters ejected from this organ were often of a bright green colour. In some instances, the fever assumed the remittent type—all the symptoms being greatly aggravated on alternate days—the intervals inspiring sanguine hopes of speedy convalescence.

"The symptoms connected with deranged cerebral function usually take the lead of all others; and even though the patient have not been seen till other organs have begun to suffer, I have generally found it expedient to prevent the tendency to congestion, and sometimes, though rarely, to inflammation, by local applications to the head. If there be no great deficiency in the general heat of the body, a cold embrocation applied after the head has been shaved is obviously agreeable to the feelings of most patients, and conduces much to the cure. Leeches or cupping, where symptoms are more urgent, are often very usefully employed to diminish the congestion of blood in the head, when general bleeding would be inadmissible. But besides the affection of the head and nervous system which seems to be connected with the first impression of fever, I am quite convinced that there is a secondary state of cerebral irritation, which depends upon the mischief going on in the intestines; and this often shows itself after the fever has continued for several days, increasing with the increase of the abdominal affection, and going on till it produces that general nervous agitation, with injected conjunctiva and constant delirium, which often closes the scene of life." 179.

In some cases, the intestinal irritation followed very quickly the first steps of the fever—generally before patients are sent to hospitals, which is not usually till after a week or more of illness. In such cases the stomach and intestinal canal had become greatly deranged—with tenderness at the epigastrium—and several watery dejections daily.

"The character of these dejections is peculiar: they are very loose, and appear as if a quantity of powdery matter of the colour of ochre were thrown into turbid water and sunk in its state of powder to the bottom. Occasionally the patient complains of pain on pretty severe pressure of the abdomen; but sometimes this is not the case. To obviate this state of the bowels, which appears to be dependent on an inflammatory condition of the mucous membrane, I find that the combination of the Hydrargyrum cum Creta, the Ipecacuanha, and the Compound Chalk Powder in different proportions, is almost always the most applicable remedy; and in many cases I have scarcely used any other combination throughout the disease. Under this treatment, simple as it may appear, with the mildest nourishment, I have seen the stools gradually change their character, the febrile symptoms regularly retire, and a state of complete convalescence succeed to the most threatening symptoms. When the tenderness of the abdomen is considerable, leeches to the amount of twelve, fourteen, or twenty, or, if the sensibility of the part does not absolutely prohibit the use of cupping-glasses, the abstraction of ten or twelve ounces of blood in that way, sometimes affords remarkable relief, more particularly if succeeded by the application of fomentations. The most alarming symptom is the irritable state of the stomach accompanied by frequent vomiting, when a quantity of green fluid is usually thrown up either spontaneously or whenever the attempt is made to administer nourishment or medicine. In this case it becomes absolutely necessary to allay the irritation of the stomach; as not only are we prevented from administering the necessary remedies and support, but the patient is completely worn out by the continuance of the painful and exhausting efforts. The task which we are here called upon to perform is often of the greatest difficulty: leeches and cupping at the pit of the stomach sometimes give very marked relief, even when the powers of the system appear much diminished; and sometimes a mustard poultice has proved beneficial, or a blister after the leeches have ceased to bleed. We may likewise have recourse to draughts, with the Subcarbonate of Magnesia and a few drops of the Vinum Opii, or a simple effervescing saline draught. Opium in the solid form with or without Calomel occasionally assists much in allaying the sickness; but often, when every thing has failed, soda water with a small quantity, not exceeding a tea-spoonful, of brandy repeated at long intervals, has remained on the stomach, and enabled it to receive and retain other things after various means have failed." 180.

In those who died at the Hospital, and where accurate examination was put in force, Dr. Bright has no hesitation in saying, that "the mucous membrane lining the ilium, (ileum) the cæcum, and the commencement of the colon, has been the chief source of that excessive irritation which has been so prominent with regard to the bowels, and that the upper part of the duodenum has probably been the source of the urgent gastric symptoms." Occasionally the whole mucous membrane of the intestines was found highly vascular "and irritated."* How far this state of things might be owing to a morbid action in the liver, as affording a vitiated, a redundant, or a defective secretion of bile, Dr. B. does not take upon him to say. The liver did not present any marked change of structure on dissection. This, however, does not prove that the function was not exceedingly deranged during life.

The most usual appearances in the mucous membrane of the bowels were those of increased action—the vascularity sometimes occurring in patches of greater or less extent, without any obvious dependence on inflammation of the mucous glands, and occasionally extending, under some form or other, through the whole tract, from the pylorus to the rectum. But this vascularity was more generally found in connexion with inflammation of the mucous glands, "which often appear like the small-pox on the second or third day of the eruption, elevated and almost transparent, and covered with minute vessels which dip into them from the lining membrane of the intestines." These glandular eminences scarcely seem to go into a state of true suppuration, but become distended with a yellow, cheesy matter, and slough off—or sometimes ulceration takes place upon their points, without any collection of yellow matter being perceptible. The same process, or nearly so, takes place both in the solitary and aggregate glands—except that, in the latter, the appearance becomes much more formidable, and the mischief more extensive. The masses or clusters of conglobate glands are chiefly placed along that part of the intestine which is furthest from the insertion of the mesentery; and when the parts are irritated by disease, three, four, or five considerable branches of vessels are seen passing on the mucous membrane, from the mesentery on each side, towards the cluster of congregate glands. These divide and subdivide before they reach the glands; and,

---

* This, we should think, is a tautology—if, indeed, it be a proper expression. Vascularity is a post-mortem evidence of irritation during life. But the fact is, irritation is a term inapplicable to dead parts.—ED.

running in part over the surface of the cluster, till their distribution is lost to the eye, they enter apparently into the thickened mass of glandular structure beneath.

The glands themselves seem first to enlarge, become quite visible, and, after some time, constitute a thick flat mass, of a lighter colour than the surrounding parts, which occasionally so spreads on the surface, that the edges overhang the base nearly the sixth of an inch. Sometimes this thickened mass is dark, as though some blood had been effused beneath the mucous coat, which is, at first, simply elevated, but not ulcerated. Dr. Burne, describing the same appearance, gives a more minute and very satisfactory explanation of it:—"These red patches have very much the appearance of spongy granulations, and have ordinarily been set down as ulcerations of the mucous membrane; and, on looking on them cursorily, one would come at once to that conclusion. They, also, give to the affected portion of intestine a preternatural density and thickness; and the thickening *tending inwards* may lead one to consider it a swollen state of the mucous membrane itself, but, by close examination, the thickening and increased density prove to be adventitious deposit in the sub-mucous tissue, and this thickening of the sub-mucous tissue elevates and stretches the mucous membrane, which stretching does away with its natural villous appearance, and makes the surface smooth and shining." 126. The surface of these thickened patches soon passes, however, into ulceration, the ulcers being, as to size and character, in proportion to the degree of irritation which they have suffered. Sometimes they are superficial and mild, at others, deep, uneven, and filled either with fæces or a dirty slough, tinged yellow with bile.

After the inflammation has subsided, and sloughing has apparently removed the greater part of the glands, the ulcers become shallow, and their margins fall in, leaving the muscular and sometimes, even, the peritoneal coat largely exposed. "This excavation is filled up by a process of granulation, which may be seen very beautifully, by suspending the intestine, cut open, before a lamp, or bright sunshine, and examining it with a common lens. The granulations are then seen, sometimes arising in broken lines around a central point; and, when the whole is healed, a scar remains visible for some time, not unlike a superficial scar from the small-pox, and generally interspersed with slight elevations, of a greyish colour. This scar *appears to be covered with a true mucous membrane, the surface being quite continuous with the membrane lining the rest of the canal.*" 182.

We have frequently examined, with the most minute attention, the mucous surface of intestines, ulceration of which had been suspected for some time previous to death; and, although we have seen patches presenting an appearance similar to that here described, we have not been able to satisfy ourselves that these had been the seat of former ulcers, much less to determine whether they were covered with a real mucous texture. Neither has Dr. Burne met "with any cicatrices, or other proofs of ulcers of the mucous membrane having healed." 129.

We believe, however, that there is nothing so peculiar about intestinal ulcers, which renders it impossible to heal them; but we conceive that it is a more rare occurrence than Dr. Bright imagines, and that, when it does occur, we have no re-production of the mucous coat, but a false surface, formed by the effusion of coagulable lymph. The simple continuity of the surface of the cicatrix with the surrounding membrane, is no proof that the structure of that surface and this membrane is the same; and, having never been successful in discovering real villous structure in such scars, we doubt much whether Dr. B. can regard continuity, in the absence of villous texture, as unequivocal evidence of the existence of mucous membrane.

The space occupied by these ulcers is very variable, but they will, in general, be found in greatest number at the lower end of the ileum, cæcum, and valve of the colon; but, as the glandular distribution is more simple in the large intestines, " the ulcers usually commence by small rounded elevations, and not in spreading masses." 188.

The peritoneal coat forming the floor of the ulcer, is usually discoloured and dark, but sometimes it is decidedly inflamed, when the abdominal symptoms become much aggravated, and, after death, sero-purulent effusion is found, accompanied with agglutination of the intestines. In very rare and severe cases, this inflammation is permitted to pass into ulceration and perforation of the peritoneum, when the contents of the intestines are extravasated into the abdominal cavity, excite general inflammation, and hasten death. When intestinal disease proceeds so far, the mesenteric glands, especially those opposite the ulcers, which will generally be found in that part of the canal most distant from the mesentery, seldom entirely escape. They are almost always enlarged and vascular, and sometimes even suppurate, so that Dr. B. has " seen them apparently on the point of discharging themselves through the peritoneum into the cavity of the abdomen; but, I believe, that not unfrequently the pus, even after it has been formed, is absorbed and *quietly* (had this expression referred to the glandular tumefaction, and not to the pus, it had been less objectionable) *subsides*." 183.

These morbid changes wrought in the intestines by fever, Dr. B. has still further illustrated by three admirably executed plates, which, if they have any defect, are, perhaps, rather too highly coloured. When ulceration takes place, and, especially, in its most confirmed stages, the mucous membrane is in general very vascular; but this vascularity does not present the vermilion vividness of an inflamed muscle. It is of a darker shade, and it is only by exposing it for some time to the air that the livid tint is displaced by a brighter red. This change, from exposure to the atmosphere, as it is considerable, should not be overlooked by the artist, since it will betray him into the use of colours overcharged for Nature, and induce him to impart a character to his drawing, for which he was indebted to an accident.

Although our author confines himself to the pathology and treatment of fever complicated with abdominal disease, he does not wish it to be understood that the intestines are the only organs under the morbid influence of this disease. The following passage corresponds with our own experience, and so supports the views of fever, inculcated in this Review, that we wish to press it upon the attention of our readers; "although I have thus minutely described the morbid appearances of the intestinal canal in certain cases of fever, yet it must be remarked that, even in these very cases, other organs suffer almost as much, and, in individual instances, much more than the intestines; particularly, the brain and its membranes evince marks of congestion; and the effusion of serum and even *fibrine* occasionally takes place; nor is it at all unusual to find symptoms of inflammation in the lungs, followed *by all the alteration of structure observed in the most acute pneumonia*." 183.

The symptoms which introduce fever, accompanied with intestinal affection, frequently differ but little from the ordinary signs. The bowels have been occasionally relaxed, but more frequently are in an opposite state, when recourse should be had to such purgatives as can efficiently remove the accumulated fæces. For this purpose, calomel, followed by castor oil, is recommended, or calomel with rhubarb may be sometimes a more convenient combination. Whichever be preferred, the frequency of their repetition should be guided by the nature and amount of the evacuations they produce. The observations here made are very important, "as long as the dejections are feculent and not too watery, and as long as they pass without pain, we shall never be doing harm by our purgatives. On the contrary, the moment that any thing like watery diarrhœa comes on, either after purging has produced irritation, or when from want of proper purging the contents of the bowels have given rise to it, we must always bear in mind that the mucous membrane is getting into the state referred to in the foregoing observations, after which every thing like brisk or irritating purging must be avoided. The moment the yellow ochrey diarrhœa has taken place, I think there can be little doubt that the intestines are either actually ulcerated, or are on the very point of ulceration; and then, in general, the irritation of the canal is of itself sufficient to prevent accumulations; and it must be our great and constant object to improve the secretion of the intestines and the connected viscera, rather than to purge actively. We must not, however, entertain a project of putting a stop to the diarrhœa; we must watch it carefully and constantly; and if we have any reason to doubt the sufficiency of the discharge, we must act gently by means of castor oil guarded with a few drops of laudanum, or by simple emollient glysters. But, in general, this, which I conceive to be the period when ulceration is commencing, is the time when the combination of the mildest mercurials, the hydrargyrum cum creta, and the compound chalk powder, with or without ipecacuanha, is administered with the greatest benefit; and it is advantageously continued till the cure is (be) complete." 185.

While the secretions of the mucous membrane are to be attended to, we are not to overlook other symptoms. If signs of increased vascularity or inflammation should appear, fomentations, leeches, and cupping, are to be cautiously employed. Antimonial remedies are disapproved of, from their tendency to increase intestinal irritation. If diaphoresis be, therefore, desirable, ipecacuanha wine is preferred; but, in many cases, all saline and diaphoretic medicines do more harm, by acting upon the mucous membrane, than good, by producing that equilibrium of the circulation for which they were intended.

Tonics are of great value, even where evidence exists of much mischief in the bowels, in all cases where the powers of life are sunk and labouring; but our author wisely cautions us against their too early and indiscriminate employment—"at the same time there is more danger to be feared from the too early use of stimulants, as long as the system is still able without their aid to support the febrile prostration, than there is risk in abstaining from stimulants a little beyond the period when they might possibly begin to act well. In a general way, the system seems capable of supporting itself for a few days under that degree of prostration which is connected with advanced ulceration of the bowels; and, although we cannot determine the exact state of the ulcers in these cases, yet we find that the action of stimulant and tonic remedies is often more certainly beneficial after that state of prostration has existed for some time, than when such remedies are administered with a view of obviating or anticipating the first symptoms of collapse; for, when administered too soon, they frequently kindle the inflammatory action with redoubled violence; and then it is that the most appalling combination of debility and nervous excitement is seen for one or two days to precede death." 186.

Dr. B. closes his observations upon fever with the detail of twenty-one cases; ten of which, having terminated fatally, he devotes to the pathology, and the remaining eleven are produced to illustrate his treatment of the disease. In the first two of the ten fatal cases the mucous membrane was highly inflamed, but not ulcerated; in the third, ulceration existed in its incipient stage; the fourth was complicated with and proved fatal from pneumonia, but the ulceration had proceeded a step beyond that exhibited in the preceding; the fifth is an illustration of the thickened and raised state of the mucous membrane from enlargement of the submucous glands, when the villous surface is stretched, smooth, and polished, "looking more like a fungus growing on the surface than an ulcer, and spreading out much wider at the top than at the base." 193. The sixth case illustrates the advanced stages of ulcerative action, where the whole mass of the aggregate glands forms one deep slough, and where the cerebral functions are sympathetically deranged;—ulceration has "dissected out" the fibres of the muscular coat in the seventh case, leaving the peritoneal alone as a partition between the intestinal and abdominal cavities;—in the eighth, the cerebral disorder, produced by the intestinal disease, becomes the immediate cause of death;—the ulcers in the ninth are supposed to have been undergoing the important process of repair, and the tenth case proved fatal by peritonitis, arising from the irritation of fæces, that escaped through an ulcer in the coats of the ileum. 200.

Of the eleven cases illustrative of Dr. Bright's treatment of intestinal fever, the first three were attended with relaxed stools, irritated bowels, red tongue, and abdominal tenderness; and gradually recovered under the use of medicines, exclusively adapted to regulate the secretions and allay the irritability of the mucous membrane; viz. pulv. cret. comp., vin. ipec., sod. subcarb. sic., ol. ricini, mist. salin. the application of leeches and fomentations to the abdomen. The fourth case is adduced to show "the excellent effects of leeches on the abdomen, and of regular and full (intestinal, we suppose) action, maintained by the mildest means, where there is reason to suppose that the internal coat of the intestines is suffering." 207. The fifth and sixth cases present nothing remarkable; the tenth was complicated with pneumonic inflammation, and the rest were suspected to have been accompanied with actual ulceration in the mucous coat, which was healed by leeches and fomentations, sinapisms and blisters, in the first stage; by hyosciamus joined to calomel, and conium to pulv. ipec. in the form of pill, hyd. c. creta with pulv. ipec. afterwards; and, during convalescence, sulph. quinæ, infus. casc. c., confect. aromat., infus. serpentarii c., subcarb. ammon. and small quantities of Port completed the cure.

After observing, that to multiply similar cases to any extent would be little better than needless repetition, convinced, as he is, that their treatment cannot be too simple, and that, when the practitioner is aware of the morbid changes going on in the intestines, he ought to be guided by symptoms, and not be deterred from *the great object* of allaying the mucous irritation, whatever other indication may require his attention; he concludes with this important caution—"There is one point of very great importance, which should likewise be carefully borne in view, and that is the great danger of relapse, unless the means are continued till the cure is completed, and the consequent necessity of continuing the use of the alterative treatment, even after the period of apparent convalescence; and, as it is quite impossible to say what ravages may have been committed by the disease on the membrane of the bowels, it is impossible to point out the time necessary for its perfect repair. I have no hesitation in saying that, in case 85, the ulceration of the bowels was not perfectly healed for many weeks, and the patient had a relapse of symptoms, with pain in the right iliac region, diarrhœa, and slightly bloody evacuations, about six weeks after he was able to go into the country as convalescent. It is our duty, in all these cases, to enjoin the strictest attention to diet, and to the state of the bowels, for, *at least*, *three months after recovery;* for it is quite consistent with what we know of this disease, to believe it possible, that for that time at least, some one of the ulcers may not be healed, and an injured portion of the peritoneum may be the only barrier between the intestinal canal and the cavity of the abdomen." p. 222.

We have now closed our review of this very valuable performance, which we can safely and unqualifiedly recommend to those whose circumstances enable them to procure it, as practical in its doctrines and orthodox in its practice, faithful to nature, and accurate in its details. Morbid anatomy is the only ground-work upon which we can rear, with success to ourselves, or safety to our patients, efficient and consistent rules for the treatment of disease. He, who is ignorant of the natural results of unhealthy action, knows not what to do, because he knows not what to dread. His practice must be timid and inert, through fear of doing harm; or bold and hazardous, from not knowing what to fear. If he cure, he cures by chance; and if his patients die, they die, but he knows neither how, nor wherefore. We hail the present, therefore, as a hopeful era in the history of medicine. Pathology is the favourite of the day, and, it is to be hoped, we are making hasty advances to that happy period, when we will be enabled, by our knowledge of symptoms, remedies, and disease, to ascribe every effect to its proper cause, to rank every appearance in its proper place, to attach to every phenomenon its proper value, to prescribe by surer rules, and to cure with greater certainty.

